# A Narrative Review of Protective Effects of Natural Compounds Against Lipopolysaccharide‐Induced Injuries

**DOI:** 10.1002/fsn3.70026

**Published:** 2025-01-31

**Authors:** Akbar Anaeigoudari

**Affiliations:** ^1^ Department of Physiology, School of Medicine Jiroft University of Medical Sciences Jiroft Iran

**Keywords:** carvacrol, crocin, lipopolysaccharide, quercetin, thymoquinone

## Abstract

Lipopolysaccharide (LPS) is a large amphipathic glycoconjugate molecule in the cell wall of Gram‐negative bacteria. This bacterial endotoxin binds to toll‐like receptor 4 (TLR4) and stimulates the inflammatory reactions and oxidative stress. The current paper presents the protective effects of natural compounds against LPS‐induced injuries. The relevant findings were extracted from PubMed, Web of Science, Scopus, and Google Scholar databases from the beginning of 2005 until the end of September 2023 were employed. The results of in vitro and in vivo studies indicated that thymoquinone, crocin, carvacrol, and quercetin effectively attenuated LPS‐induced damages via lowering the level of inflammatory cytokines and free radicals. These natural compounds could also amplify the antioxidant defense against LPS by increasing the activity of antioxidant enzymes such as superoxide dismutase (SOD) and catalase (CAT). In addition, a part of the protective effects of these phytochemicals against detrimental impacts of LPS is attributed to their ability to downregulate the TLR4 expression and to inhibit the NF‐κB signaling pathway. Briefly, the protective effects of natural compounds mentioned in current review against LPS‐caused damages mainly are mediated by their anti‐inflammatory and antioxidant activities.

## Graphical abstract

Inflammation and oxidative stress induced by LPS results in organs dysfunction. Natural compounds such as thymoquinone, crocin, carvacrol and quercetin inhibit the detrimental effects of LPS.

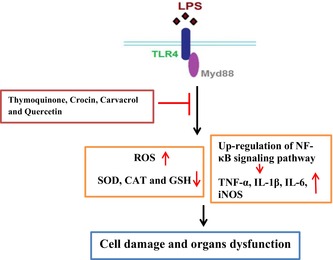

## 1 Introduction

Toxins are the pathogenicity factors generated by bacteria that threaten human health (Mirza, Walhout, and Ambros 2023). Lipopolysaccharide (LPS) is a bacterial endotoxin presented in the outer leaflet of the outer membrane of Gram‐negative bacteria (Anaeigoudari et al. 2015). LPS possesses three parts, including lipid A, O‐antigen repeats, and core polysaccharides. The toxic effects resulting from LPS are attributed to lipid A (Wang and Quinn 2010). LPS is synthesized in the cytoplasm and then is exported on the surface of the bacterial cell wall. Toll‐like receptor 4 (TLR4) is a receptor expressed on the surface of various immune cells such as neutrophils, macrophages, and monocytes. LPS binds to TLR4 and induces intracellular signaling pathways (Chang et al. 2023; Xie et al. 2023). After recognition by LPS, TLR4 activates adaptor proteins such as Trif, Tram, and MyD88 and triggers intracellular protein kinases (Yamamoto et al. 2003). It has been found that TLR4 uses Trif, Tram, and MyD88 to activate the mitogen‐activated protein kinase (MAPK) and nuclear factor κB (NF‐κB) signaling pathways, leading to the release of the inflammatory cytokines such as interleukin (IL) ‐1 (Harari et al. 2006). In high concentration, LPS induces fever and septic shock and disturbs the function of the heart, kidney, and lung (Wang and Quinn 2010). Furthermore, LPS induces the excessive production of inflammatory cytokines and oxidative stress, causing the organs to dysfunction (Hosseini et al. 2018). Considering this subject, LPS is widely used to induce systemic and central inflammation in experimental studies (Anaeigoudari, Soukhtanloo, Reisi, et al. 2016; Anaeigoudari, Soukhtanloo, Shafei, et al. 2016). It has been recognized that peripheral administration of LPS impaired synaptic plasticity and spatial memory by increasing the tumor necrosis factor‐α (TNF‐α), malondialdehyde (MDA), and nitric oxide (NO) metabolites level and decreasing thiol content and superoxide dismutase (SOD) and catalase (CAT) activity in the hippocampus tissue of rats (Anaeigoudari, Soukhtanloo, Reisi, et al. 2016; Anaeigoudari, Soukhtanloo, Shafei, et al. 2016). In addition, inflammation and oxidative damage caused by systemic injection of LPS have been shown to disturb the normal function of the liver (Beheshti et al. 2021), heart (Yuxuan et al. 2023), and kidney (Yi et al. 2023).

Phytochemicals have been demonstrated to have beneficial effects against different diseases (Anaeigoudari, Anaeigoudari, and Kheirkhah‐vakilabad 2023). Thymoquinone, crocin, carvacrol, and quercetin are phytochemicals with anti‐inflammatory properties (Adinew et al. 2023; Aslani et al. 2023; Cui et al. 2022; Javanbakht et al. 2023; Marinelli et al. 2022). Thymoquinone is a monoterpene obtained from *Nigella sativa* with neuroprotective (Baghcheghi et al. 2018), anticonvulsant (Hyder Pottoo et al. 2022), antianxiety (Gilhotra and Dhingra 2011), antidepressant (Anaeigoudari 2022), and anticancer (Selmi et al. 2023) activities. Crocin isolated from saffron also exerts positive effects on learning and memory (Hassani et al. 2020), as well as cardiovascular (Shafei et al. 2017) and gastrointestinal (Xie et al. 2019) systems. Carvacrol is a phenolic compound extracted from the essential oil of oregano and thyme that exhibits antimicrobial (Mączka et al. 2023) and antitumor (Arunasree 2010) activities. Quercetin is a flavonoid compound that possesses a wide range of biological impacts, including antihypertensive (Elbarbry et al. 2020), anti‐obesity (Su et al. 2022), and anti‐hyperlipidemia (Hosseini et al. 2021) activities. The present review focuses on the therapeutic effects of phytochemicals, including thymoquinone, crocin, carvacrol, and quercetin against LPS‐ induced injuries in in vitro and in vivo studies. This paper also highlights the anti‐inflammatory and antioxidative mechanisms evoked by these compounds against the detrimental effects of LPS.

## 2 Method

The findings of the present review were collected by exploring the scientific databases, including Web of Science, PubMed, Scopus, and Google Scholar. The relevant data were extracted from the beginning of 2005 until the end of September 2023. Key words included “lipopolysaccharide” and “thymoquinone” or “crocin” or “carvacrol” and “quercetin”.

## 3 Protective Effects of Thymoquinone

### 3.1 In Vivo

Inflammation is a body's defense reaction by which the immune cells recognize and neutralize the detrimental and foreign agents. Inflammation is categorized into acute and chronic phases. Acute inflammation is a fast response of the immune system against harmful stimuli such as toxins and can last for a few days. Chronic inflammation is a slow and long‐term reaction of the immune system against noxious stimuli and may last for several months (Ferrero‐Miliani et al. 2007). Toll‐like receptors (TLRs) play a fundamental role in response to the microbial toxins (Avila et al. 2012). LPS binds to the TLR‐4 and stimulates the nuclear factor κB (NF‐κB) signaling pathway, causing the release of inflammatory mediators (Zou et al. 2023). Natural compounds can alleviate the bacterial infections‐induced inflammation (Arab et al. 2023; Liu et al. 2021). In an animal study, the impact of a single dose (2 mg/kg) of nanoparticles of thymoquinone was checked on chronic lung injuries resulting from intratracheal installation of LPS. In this study, the Wistar rats were exposed to LPS twice weekly during 8 weeks, and nanoparticles of thymoquinone were used after the last dose of LPS. The histopathological findings indicated that repetitive intratracheal installation of LPS induced collapse of the alveoli, pulmonary edema, obstruction of the airways, and pulmonary fibrosis. Nanoparticles of thymoquinone significantly alleviated these histopathological alterations. This improving effect of nanoparticles of thymoquinone coincided with a decreased serum level of IL‐10 and TGF‐β1 (Saghir et al. 2021). Boskabady et al. also examined the thymoquinone effect on lung injuries in the rats exposed to 1 mg/kg of LPS. LPS was injected intraperitoneally for two weeks, and thymoquinone (5 and 10 mg/kg) was administered 30 min before LPS. Then, lung pathological changes, white blood cell (WBC) count in the blood and bronchoalveolar fluid (BALF), and INF‐ɣ, TGF‐β1 and PGE2, and IL‐4 concentration in the BALF were monitored. The results showed that positive therapeutic effects of thymoquinone on LPS‐stimulated lung pathological changes were associated with a significant mitigation in WBC count and the level of INF‐ɣ, TGF‐β1, and PGE2 and a remarkable enhancement in the concentration of IL‐4 (Boskabady et al. 2021). In another study, the effectiveness of 3 mg/kg of thymoquinone against LPS‐caused lung tissue destruction in BALB/C male mice was evaluated. In this study, the use of thymoquinone 1 h before intratracheal injection of LPS had a remarkable recovery in neutrophil migration, intra‐alveolar hemorrhage, and alveolar demolition (Colak et al. 2020). Intraperitoneal injection of thymoquinone at a dose of 1 mg/kg also prevented the destructive effect of LPS on pulmonary blood vessels in the rats by restoring the pathological damages and modulating the level of inflammatory factors such as IL‐1β and TNF‐α (Al‐Gabri et al. 2019).

Scientific evidence exhibited that use of LPS for a mild term period disturbs learning and memory processes in the rodents. In the rats receiving 1 mg/kg of LPS for two weeks, thymoquinone administration (2, 5, and 10 mg/kg) 30 min before LPS significantly ameliorated spatial and non‐spatial memory in Morriss Water Maze (MWM) and passive avoidance tasks. Biochemical analysis also demonstrated that the hippocampal tissue level of TNF‐α, IL‐6, MDA, and NO was lower, and total thiol concentration and SOD and CAT activity were higher in the rats treated with thymoquinone than those of the LPS group (Bargi et al. 2017). In the male Wistar rats treated with 1 mg/kg of LPS for three weeks, thymoquinone at 2, 5, and 10 mg/kg doses also could improve myocardial fibrosis through decreasing the cardiac level of IL‐1β, TNF‐α, NO, and MDA, and enhancing the total thiol content and SOD and CAT activity (Asgharzadeh et al. 2018). Furthermore, the protective effect of thymoquinone (2, 5, and 10 mg/kg) on LPS‐induced hepatotoxicity in the male Wistar rats was documented by reducing the MDA concentration and increasing the total thiol content and SOD and CAT activity (Asgharzadeh et al. 2017). The precise mechanism of the anti‐inflammatory activity of thymoquinone on LPS‐caused inflammation is not clear. It has been recommended that thymoquinone can suppress the activity of lipoxygenase and cyclooxygenase (COX) pathways. COX‐2 is a form of COX that mediates inflammation (Mezayen et al. 2006). It is suggested that the protective effects of thymoquinone against LPS‐induced damages may be mediated by mechanisms that targets COX‐2‐related inflammatory factors synthesis.

### 3.2 In Vitro

RBL‐2H3 cells were exposed to 0.1 μg/mL of LPS for 12 h with and without the presence of 10 μM of thymoquinone. Then gene transcription of IL‐5, IL‐13, IL‐10, GATA, c‐Fos, c‐ Jun, and phospho‐c‐Jun was assessed. It was recognized that thymoquinone effectively suppressed LPS‐triggered gene expression of IL‐5, IL‐13, and GATA‐2. However, thymoquinone could not affect the production of IL‐10, c‐Fos, c‐ Jun, and phospho‐c‐Jun (El Gazzar et al. 2007). The combined effect of *Nigella sativa* oil (55, 110, 220, 440 μg/mL) and thymoquinone (4.5, 9.0, 18.0, 36.0 μM) on Human Coronary Artery Endothelial Cells (HCAECs) treated with 200 μg/mL of LPS for 24 h was also checked. The results revealed that thymoquinone could reduce the expression of biomarkers such as Intercellular Adhesion Molecule‐1 (ICAM‐1), and Vascular Cell Adhesion Molecule‐1 (VCAM‐1) in a dose‐dependent manner (Khan et al. 2023). The effects of thymoquinone have been collected in Table 1.

**TABLE 1:** Protective effects of thymoquinone.

Type of study	Doses	Organ/cell	Effects	References
In vivo	2 mg/kg	Lung	Alleviation of histopathological alterations and reduction of IL‐10 and TGF‐β1level	Saghir et al. (2021)
In vivo	5 and 10 mg/kg	Lung	Mitigation of WBC count and the level of INF‐ɣ, TGF‐β1, and PGE2 and enhancement of the concentration of IL‐4	Boskabady et al. (2021)
In vivo	3 mg/kg	Lung	Modulation of neutrophil migration, intra‐alveolar hemorrhage, and alveolar demolition	Colak et al. (2020)
In vivo	1 mg/kg	Lung	Suppression of the effect of LPS on pulmonary blood vessels in rats by restoring pathological damages and modulating the level of inflammatory factors such as IL‐1β and TNF‐α	Al‐Gabri et al. (2019)
In vivo	2, 5, and 10 mg/kg	Brain	Amelioration of spatial and non‐spatial memory, and reduction of the hippocampal tissue level of TNF‐α, IL‐6, MDA, and NO, and enhancement of total thiol concentration and SOD and CAT activity	Bargi et al. (2017)
In vivo	2, 5, and 10 mg/kg	Heart	Improvement of myocardial fibrosis through decreasing cardiac level of IL‐1β, TNF‐α, NO, MDA and enhancing total thiol content and SOD and CAT activity	Asgharzadeh et al. (2018)
In vivo	2, 5, and 10 mg/kg	Liver	Alleviation of LPS‐induced hepatotoxicity by reducing MDA concentration and increasing total thiol content and SOD and CAT activity	Asgharzadeh et al. (2017)
In vitro	10 μM	RBL‐2H3 cells	Suppression of LPS‐triggered gene expression of IL‐5, IL‐13, and GATA‐2	El Gazzar (2007)
In vitro	4.5, 9, 18, 36 μM	HCAEC cells	Decrease of the expression of biomarkers such as ICAM‐1 and VCAM‐1	Khan et al. (2023)

## 4 Protective Effects of Crocin

### 4.1 In Vivo

Acute respiratory distress syndrome (ARDS) is a life‐threatening lung disease that is characterized by damage of lung endothelial cells and accumulation of fluid into lung tissue (Kwok et al. 2023). It has been recognized that the distraction of endothelial glycocalyx and inflammatory reactions have a key role in the pathogenesis of ARDS (Huang et al. 2018). In the LPS‐induced mice model of ARDS, the animals were distributed into control, LPS, and corcin + LPS groups. Treatment with crocin was carried out at 15, 30, and 60 mg/kg doses before LPS for 7 days. Histological assessment indicated that lung structural injuries significantly decreased in mice treated with crocin when it was compared with the LPS group. In addition, crocin amplified the integrity of endothelial glycocalyx and attenuated the activity of NFκB and mitogen‐activated protein kinase pathways (Zhang et al. 2020). The neuroprotective role of crocin against memory impairment resulting from LPS administration was evaluated by MWM and passive avoidance tests in the male Wistar rats. Besides behavioral tests the level of IL‐1β, lipid peroxidation, and total thiol were measured in the hippocampus and cortex tissues of the rats. It was recognized that treatment with 50 and 100 mg/kg of crocin shortened the time latency for capturing the hidden platform in the MWM test and increased step‐through latency in the passive avoidance test. However, researchers did not observe significant changes in the level of biochemical indicators in the brain tissue (Azmand and Rajaei 2021).

Plasminogen activator inhibitor‐1 (PAI‐1) is a protein that inhibits the plasminogen activator in plasma and attenuates fibrinolysis (Geng et al. 2023). In the female Wistar rats, LPS at a 500 μg/kg dose decreased the platelet blood counts and increased the PAI‐1 concentration. Crocin administration (10 and 100 mg/kg) 30 min before LPS prevented the decline of platelet blood counts and recovered the PAI‐1 level in the liver and the brain. In addition, crocin suppressed the accumulation of fibrin in the kidney glomeruli of the rats (Tsantarliotou et al. 2019).

### 4.2 In Vitro

Microglia cells are resident immune cells in the brain (Bai et al. 2023). Under basal conditions, they play a supporting role in synaptogenesis through producing the neurotrophic factors (De Moura et al. 2022). Activated microglia cells also can produce and release pro‐inflammatory cytokines, causing oxidative stress (Sheu et al. 2023). Meanwhile, activation of the C‐X3‐C motif chemokine receptor 1 (CX3CR1) is considered a regulating factor of the microglial cell activity. Animal studies have confirmed that CX3CR1 can suppress the activity of microglia (Deng et al. 2021). In a study, the BV2 microglial cells were treated with 0.1, 0.5, 1, 5, and 10 μM doses of crocin for 2 h prior to LPS (11 μg/mL). The expression of inflammatory markers and CX3CR1 was detected by the relevant molecular techniques. It was found that crocin dose‐dependently could mitigate the expression of IL‐1β, IL‐6, TNFα, iNOS, and COX‐2 in microglial cells challenged by LPS. Inhibition of the NF‐κB/YY1 signaling pathway by crocin also led to increased expression of CX3CR1 (Lv et al. 2016). In the study handled by Rahim et al. H9c2 cells received crocin at 10, 20, and 40 μM doses for 24 h, and then they were treated by LPS for another 24 h. Based on the results of the study, crocin exerted its anti‐toxicity effects against LPS via lessening the concentration of IL‐1β, IL‐6, TNF‐α, and NO and down‐regulating the expression of COX‐2 and iNOS (Rahim et al. 2019). The effects of crocin have been illustrated in Table 2.

**TABLE 2:** Protective effects of crocin.

Type of study	Doses	Organ/cell	Effects	References
In vivo	15, 30, and 60 mg/kg	Lung	Improvement of lung structural injuries, amplification of the integrity of endothelial glycocalyx, and attenuation of the activity of NFκB and mitogen‐activated protein kinase pathways	Zhang et al. (2020)
In vivo	50 and 100 mg/kg	Brain	Decrease of the time latency for capturing the hidden platform in the MWM test and increase of step‐through latency in the passive avoidance test	Azmand and Rajaei (2021)
In vivo	10 and 100 mg/kg	Liver and Brain	Increase of platelet blood counts, improvement of the PAI‐1 level in the liver and the brain, and reduction of fibrin concentration in the kidney glomeruli	Tsantarliotou et al. (2019)
In vitro	0.1, 0.5, 1, 5, and 10 μM	BV2 Microglia cells	Mitigation of IL‐1β, IL‐6, TNFα, iNOS, and COX‐2 expression and up‐regulation of CX3CR1 expression by inhibiting NF‐κB/YY1 signaling pathway	Lv et al. (2016)
In vitro	10, 20, and 40 μM	H9c2	Reduction of IL‐1β, IL‐6, TNF‐α, and NO concentration and down‐regulation of COX‐2 and iNOS expression	Rahim et al. (2019)

## 5 Protective Effects of Carvacrol

### 5.1 In Vivo

Neuro inflammation followed by systemic inflammation has been demonstrated to be a risk factor causing sickness behaviors (O'Neill et al. 2021). Neuroprotective impacts of different concentrations of carvacrol were figured out using an animal model of systemic inflammation induced by 1 mg/kg of LPS. For this purpose, carvacrol was injected at 25, 50, and 100 mg/kg doses 30 min prior to LPS. In the forced swimming test, the groups receiving carvacrol had less immobility time and more climbing time than the LPS group. Time spent and entries in open arms were longer in the LPS‐carvacrol groups versus the LPS group in the elevated plus maze test. The results of the open field test also exhibited that all three doses of carvacrol increased crossing, time spent, and traveled distance by the rats in the central zone of the apparatus. In order to confirm the anti‐inflammatory and antioxidative properties of carvacrol against neuroinflammation caused by LPS, the researchers measured the level of TNF‐α, NO, MDA, and thiol in the brain tissue of the animals. It became clear that all doses of carvacrol lowered the level of TNF‐α and NO in the brain. Carvacrol at 50 and 100 mg/kg doses reduced the MDA concentration, and at a 100 mg/kg dose, elevated the thiol level in the brain of animals (Salmani et al. 2022). Amooheydari et al. started carvacrol administration at 25 and 50 mg/kg doses two weeks before the LPS (1 mg/kg) injection and then repeated it for days 15–19 simultaneously with LPS. The findings extracted from this study indicated that carvacrol at 25 mg/kg dose recovered the spatial memory deficits caused by LPS. This neuroprotective effect of carvacrol is associated with the decreased level of TNF‐α and inhibition of lipid peroxidation in hippocampus and cortex tissues. In this study, a high dose of carvacrol (50 mg/kg) was not effective in preventing the harmful impacts of LPS on the brain. It has been propounded that the polyphenols, such as carvacrol, in high doses have prooxidant activities. Therefore, they may apply reverse dose–response effects (Amooheydari et al. 2022). In male Sprague–Dawley rats, memory impairment was induced by injection of 2 μL/min of LPS‐containing solution into the lateral ventricle of the brain. Daily injection of carvacrol at 25, 50, and 100 mg/kg doses for 21 days alleviated the destructive effects of LPS on recognition, discrimination, and spatial memory via down‐regulating the level of IL‐1β, TNF‐α, COX‐2, and TLR4 and up‐regulating the mRNA expression of brain‐derived neurotrophic factor (BDNF) in the hippocampus and prefrontal cortex tissues of the rats (Lee et al. 2020b). Cognitive deficits followed by injection of 1 mg/kg of LPS also were reversed by carvacrol when it was employed intraperitoneally in 25, 50, and 100 mg/kg doses 30 min prior to LPS. These memory‐enhancing effects of carvacrol were also referred to its anti‐inflammatory and anti‐oxidative activities (Hakimi et al. 2020). Anticonvulsive properties of carvacrol at 100 mg/kg dose on LPS‐evoked seizures were confirmed. In an experimental work, male Wistar rats were exposed to LPS at a 400 μg/kg dose 4 h before PTZ, and carvacrol was administered immediately after LPS. The seizure behaviors and expression of COX‐1 and COX‐2 in the hippocampus tissue of the rats were checked. Modulation of LPS‐ caused seizures by carvacrol was associated with a reduced level of COX‐1 and COX‐2 expression (Sadegh and Sakhaie 2018).

Anti‐inflammatory activity of carvacrol led to the treatment of LPS‐caused acute lung injuries in mice. In this study, administration of carvacrol at 20, 40, and 80 mg/kg before LPS for seven days, inhibited the NF‐κB and MAPKs signaling pathways and postponed the IL‐1β, IL‐6, and TNF‐α production in the lung tissue of the mice (Feng and Jia 2014). The hepatoprotective effect of carvacrol against LPS also has been documented by Mortazavi et al. Rats were treated with carvacrol at 25, 50, and 100 mg/kg doses 30 min prior to LPS (1 mg/kg) for two weeks. Carvacrol overturned the LPS impacts on the liver of rats by decreasing the level of aspartate transaminase (AST), alanine aminotransferase (ALT), alkaline phosphatase (ALK‐P), MDA, NO, and IL‐6 and increasing the level of total protein, albumin, thiol, SOD, and CAT (Mortazavi et al. 2021). The intestinal protective effect of carvacrol supplementation against LPS was evaluated. In an animal study, 25 g/t of carvacrol was added to the basal diet of the male and female rabbits exposed to 200 μg/kg of LPS for 28 days. It was clear that feeding with carvacrol alleviated the anxious effects of LPS on the ileum and cecum of rabbits via downregulating the inflammatory cytokines production, including TNF‐α, IL‐1β, IL‐6, and IL‐8. It has been speculated that the anti‐inflammatory activities of carvacrol may be carried out by stopping the TLRs/NF‐κB/TNF‐α/MAPK pathway (Wu et al. 2023).

### 5.2 In Vitro

In an in vitro study, treatment with 6.25 and 50 μM doses of carvacrol for 24, 48, and 72 h exerted the anti‐inflammatory effects on HL‐1 cardiomyocytes exposed to 5 μg/mL doses of LPS. Inflammatory and oxidative stress analyses showed that both doses of carvacrol downregulated the expression of TLR‐4, NALP3, NFκB, IL‐1β, and ROS by LPS‐stimulated HL‐1 cells (Marconi et al. 2022). The protective effects of carvacrol have been displayed in Table 3.

**TABLE 3:** Protective effects of carvacrol.

Type of study	Doses	Organ/cell	Effects	References
In vivo	25, 50, and 100 mg/kg	Brain	Improvement of behavioral responses, decrement of the TNF‐α, MDA, and NO concentration, and enhancement of the total thiol groups level	Salmani et al. (2022)
In vivo	25 and 50 mg/kg	Brain	Amelioration of spatial memory, decrease of TNF‐α and inhibition of lipid peroxidation	Amooheydari et al. (2022)
In vivo	25, 50, and 100 mg//kg	Brain	Amplification of spatial memory via downregulating the level of IL‐1β, TNF‐α, COX‐2, and TLR4 and upregulating the mRNA expression of BDNF	Lee et al. (2020a)
In vivo	25, 50, and 100 mg/kg	Brain	Reinforcement of memory and inhibition of inflammation and oxidative stress	Hakimi et al. (2020)
In vivo	100 mg/kg	Brain	Relief of seizure attacks and inhibition of COX‐1 and COX‐2 activity	Sadegh and Sakhaie (2018)
In vivo	20, 40, and 80 mg/kg	Lung	Suppression of NF‐κB and MAPK signaling pathways activity, and decrease of TNF‐α‐ IL‐1β and IL‐6 production	Feng and Jia (2014)
In vivo	25, 50, and 100 mg/kg	Liver	Decrease of the level of AST, ALT, ALK‐P, MDA, NO, and IL‐6 and increase of the level of total protein, albumin, thiol, SOD, and CAT	Mortazavi et al. (2021)
In vivo	25 g	Intestinal	Downregulation of the inflammatory cytokines production, including TNF‐α, IL‐1β, IL‐6, and IL‐8	Wu et al. (2023)
In vitro	6.25 and 50 μM	HL‐1	Downregulation of TLR‐4, NALP3, NFκB, IL‐1β and ROS expression	Marconi et al. (2022)

## 6 Protective Effects of Quercetin

### 6.1 In Vivo

It has been demonstrated that flavonoids possess the anxiolytic properties (Wang et al. 2023). Quercetin is a flavonoid compound that exhibits anxiolytic effects due to its anti‐inflammatory and antioxidant effects (Samad et al. 2018). In an animal study, anxiety‐like behaviors were induced using the injection of 2 μL/min of LPS into the lateral ventricle of the male rats. Quercetin was also administered at 10, 50, and 100 mg/kg doses 21 days after LPS administration. It was recognized that daily injection of quercetin effectively recovered anxiety‐like behaviors in the rats when they were checked by the elevated plus maze test and the open field test. In this study, quercetin also reduced the brain levels of IL‐1β, IL‐6, COX‐2, NFκB, and iNOS and increased the level of BDNF (Lee et al. 2020b). Evaluation of sickness behaviors using forced swimming, splash, and open field tests demonstrated that quercetin injection at 50 mg/kg dose for seven days could alleviate the depressive symptoms evoked by LPS (0.83 mg/kg) in the male rats. This therapeutic effect of quercetin was related to the decreased level of inflammatory cytokines, NFκB, and iNOS in the hippocampus and prefrontal cortex of the rats (Adeoluwa et al. 2023). Copine 6 is a protein that can regulate synaptic plasticity (Burk et al. 2018). The triggering receptors expressed on myeloid cells (TREM) are a family of cell receptors that their expression alters in inflammatory reactions (Zhang et al. 2023). TREM‐1 is an activating receptor expressed on leukocytes such as monocytes and neutrophils that has a vital role in the occurrence of inflammation (Yue et al. 2023). TREM‐2 has been also suggested to evoke the inflammatory responses in inflammatory bowel disease (IBD) by affecting the function of dendritic cells (Natale et al. 2019). Treatment of the rats exposed to 5 mg/kg of LPS by 40 mg/kg of quercetin resulted in a significant improvement in behavioral indexes in the saccharin preference test, the Y maze test, and the forced swimming test. However, this dose of quercetin exerted no significant impact on behavioral parameters in the MWM test and the level of IL‐6, C‐reactive protein, and nesfatin‐1. The results showed that quercetin also could balance the brain level of BDNF, P‐TrkB/TrkB, synapsin 1, copine 6, TREM‐1, and TREM‐2 in the LPS‐challenged rats (Fang et al. 2020). The ability of 200 mg/kg of quercetin to ameliorate LPS‐caused intestinal oxidative stress in broiler chickens has been documented. In this study, quercetin was mixed in the basal diet of the broiler chickens, and LPS was administered at a dose of 0.5 mg/kg. According to the results, the antioxidant effects of quercetin against LPS were associated with the reduced level of MDA, 8‐hydroxy‐2′‐deoxyguanosine (8‐OHdG), and enhanced concentration of SOD and glutathione peroxidase (GSH‐Px). Quercetin also could upregulate the Nrf2 level, suggesting that quercetin alleviates the LPS‐stimulated intestinal oxidative damage via activating the Nrf2 signaling pathway (Sun et al. 2020). Huang et al. examined the effect of oral administration of 50 mg/kg of quercetin to protect LPS‐challenged lungs in the rats. The histopathological and biochemical results illustrated that pretreatment with quercetin could attenuate the destructive effect of LPS on the lung tissue as well as decrease the BALF protein level and neutrophil count, and MDA concentration and increase the activity of SOD, CAT, and GSH‐Px (Huang, Zhong, and Wu 2015). In LPS‐sensitized mice, intratracheal injection of 1 μM of quercetin decremented the activity of matrix metalloproteinase (MMP)‐9 and inflammatory mediators including IL‐1β, IL‐6, and TNF‐α in BALF cells (Takashima et al. 2014).

### 6.2 In Vitro

The findings of oxidative stress and inflammatory indicators indicated that quercetin at 100 μg/mL dose rescued the bovine mammary epithelial cells from damage of 1 μg/mL of LPS. This protective effect of quercetin was linked to its ability in downregulating the mRNA expression of inflammatory mediators such as IL‐1β, IL‐6 and TNF‐α and chemokines including CXCL2, CXCL5, CCL5, and CXCL8 (Jiang et al. 2022). The potency of quercetin at 5, 10, 50, 100, 200 and 400 μM doses on viability, proliferation, and apoptosis of human oral keratinocytes treated by 100 ng/mL of LPS was assessed. The results demonstrated that quercetin prevented LPS‐caused reduction in viability and increase in apoptosis of human oral keratinocytes. In addition, quercetin mitigated the level of miR‐22 and enhanced the activity of PI3K/AKT and JAK1/STAT3 pathways in human oral keratinocytes challenged by LPS (Wang et al. 2020). The cell viability, apoptosis, and inflammatory reactions of the human nasal epithelial cells in the presence of 1 μg/mL of LPS and quercetin at 10, 100, and 200 μM doses also were evaluated. It was concluded that quercetin in a dose‐dependent manner could inhibit the LPS impacts on the reduction of cell viability, apoptosis, and inflammatory reactions. These positive effects of quercetin were attributed to its ability in suppressing the miR‐21/DMBT1/NF‐κB pathway (Cheng, Luo, and Chen 2022). The protective effects of quercetin have been exhibited in Table 4.

**TABLE 4:** Protective effects of quercetin.

Type of study	Doses	Organ/cell	Effects	References
In vivo	10, 50, and 100 mg/kg	Brain	Alleviation of anxiety‐like behaviors, reduction of the brain level of IL‐1β, IL‐6, COX‐2, NFκB, and iNOS, and increase of the level of BDNF	Lee et al. (2020b)
In vivo	50 mg/kg	Brain	Improvement of depressive symptoms and decrease of NFκB and iNOS in the hippocampus and prefrontal cortex	Adeoluwa et al. (2023)
In vivo	40 mg/kg	Brain	Improvement of behavioral indexes and modulation of the brain level of BDNF, P‐TrkB/TrkB, synapsin 1, copine 6, TREM‐1, and TREM‐2	Fang et al. (2020)
In vivo	200 mg/kg	Intestine	Reduction of the level of MDA, 8‐OHdG, and enhancement of the concentration of SOD and GSH‐Px, and upregulation of Nrf2 expression	Sun et al. (2020)
In vivo	50 mg/kg	Lung	Decrease of BALF protein level, neutrophil count, and MDA concentration, and increase the activity of SOD, CAT, and GSH‐Px	Huang, Zhong, and Wu (2015)
In vivo	1 μM	Lung	Decline of the activity of MMP‐9 and inflammatory mediators, including IL‐1β, IL‐6, and TNF‐α, in BALF cells	Takashima et al. (2014)
In vitro	100 μg/mL	Bovine mammary epithelial cells	Downregulation of inflammatory mediators such as IL‐1β, IL‐6, and TNF‐α and chemokines including CXCL2, CXCL5, CCL5, and CXCL8	Jiang et al. (2022)
In vitro	5, 10, 50, 100, 200, and 400 μM	Human oral keratinocytes	Mitigation of the level of miR‐22 and enhancement of the activity of PI3K/AKT and JAK1/STAT3 pathways	Wang et al. (2020)
In vitro	10, 100, and 200 μM	Human nasal epithelial cells	Increase of cell viability and suppression of apoptosis, inflammatory reactions, and the miR‐21/DMBT1/NF‐κB pathway	Cheng, Luo, and Chen (2022)

## 7 Conclusion

The results of in vivo and in vitro studies discussed in the present review demonstrate that thymoquinone, crocin, carvacrol, and quercetin attenuated the noxious impacts of LPS by suppressing the production of inflammatory mediators such as TNFα, IL‐1β, and IL‐6, and oxidative stress indexes including MDA and NO. Reports also show that potentiation of antioxidant defense via increasing the activity of antioxidant enzymes, including SOD,CAT, and GSH, by these phytochemicals contributes to protecting the cells against LPS‐caused injuries. Furthermore, it has been recognized that down‐regulation of TLR‐4 expression and inhibition of NF‐κB signaling pathway activity by these natural compounds play a key role in alleviating the adverse effects of LPS. Despite all this evidence, the molecular mechanisms are still unclear. Therefore, further studies can be carried out to elucidate these mechanisms.

## Author Contributions

**Akbar Anaeigoudari:** data curation (equal), methodology (equal), writing – review and editing (equal).

## Conflicts of Interest

The author declares no conflicts of interest.

## Data Availability

The data used in this study are available on request from the corresponding author.
